# Effects of different exercise modalities on lipid profile in the elderly population: A meta-analysis

**DOI:** 10.1097/MD.0000000000033854

**Published:** 2023-07-21

**Authors:** Hezhang Yun, Wenbo Su, Haotian Zhao, Huixin Li, Zhongjie Wang, Xianyou Cui, Changjin Xi, Ruirui Gao, Yaowei Sun, Chang Liu

**Affiliations:** a The Public Sports Department of the School, Zhejiang Guangsha Vocational and Technical University of construction, Dongyang, China; b School of Sport Science, Beijing Sport University, Beijing, China; c Department of Sports Teaching and Research, Lanzhou University, Lanzhou, China; d Department of Physical Education, Jiangnan University, Wuxi, China; e School of Journalism and Communication, Beijing Sport University, Beijing, China; f Yufeng experimental school, Kunshan, China.

**Keywords:** aged, dyslipidemias, exercise therapy, lipids, meta-analysis

## Abstract

Dyslipidemia, characterized by elevated total cholesterol (TC), triglycerides, and low-density lipoprotein cholesterol (LDL-C), as well as decreased high-density lipoprotein cholesterol (HDL-C), has long been recognized as a major risk factor for age-related diseases. Recent studies have shown that exercise can be effective in mitigating dyslipidemia in older adults. The aim of this study was to systematically evaluate the effects of different exercise modalities (aerobic exercise [AE], resistance exercise [RE], aerobic+resistance exercise [AE+RE], and high-intensity interval training) on lipid profile in the elderly population, in order to identify the most effective exercise modality for maintaining lipid profile health in older adults.

**Methods:**

A comprehensive database search of PubMed, EBSCO, Web of Science, China National Knowledge Infrastructure, and Wanfang database. Eligible studies were individually assessed according to the Cochrane Risk of Bias Tool version 2, and the data were analyzed and processed using RevMan 5.4.1 analysis software.

**Results:**

This study identified 20 randomized controlled trials with a total of 988 subjects, of which 541 were in the exercise group and 447 in the control group. Our analysis showed that AE significantly reduced TC (*P* < .05), triglycerides (*P* < .01), and LDL-C (*P* < .01), while increasing HDL-C (*P* < .01) in the elderly population. RE significantly reduced the elevation of TC (*P* < .01) and LDL-C (*P* < .05) indices in the elderly population, while AE+RE had a significant effect on decreasing TC (*P* < .01) and LDL-C (*P* < .01) indices.

**Conclusion:**

Our analysis indicates that AE is the most effective exercise modality for reducing dyslipidemia in older adults, compared to RE, AE+RE, and high-intensity interval training. These findings suggest that AE should be promoted as an important lifestyle intervention to improve lipid profile health in the elderly population. However, further research is needed to investigate the optimal duration, frequency, and intensity of AE required to achieve the most beneficial effects on lipid profile health in older adults.

## 1. Introduction

Dyslipidemia refers to an abnormal lipid profile in the bloodstream. In the elderly population, hyperlipidemia is the predominant form of dyslipidemia, characterized by elevated levels of blood lipids. This condition is considered a pathogenic risk factor for cardiovascular disease. Hyperlipidemia is typically marked by elevated levels of total cholesterol (TC), low-density lipoprotein cholesterol (LDL-C), and triglycerides (TG), as well as decreased levels of high-density lipoprotein cholesterol (HDL-C).^[1]^ Recent studies have indicated that these lipid profile abnormalities can facilitate the infiltration of cholesterol into the arterial walls, leading to deposition and accumulation, and promoting the proliferation of smooth muscle cells and fibroblasts in the arterial intima. This process can ultimately result in the development of atherosclerosis, coronary heart disease, and other cardiovascular and cerebrovascular diseases.^[2]^ Furthermore, hyperlipidemia has been associated with the development of other diseases, such as polycystic ovary syndrome, rheumatoid arthritis, and Alzheimer’s disease.^[3–5]^

With the continuous growth of the elderly population, China has now become the country with the largest number of elderly people in the world. According to the projections, by 2050, the population over the age of 65 will account for more than 26.1% of the global population,^[6]^ with the number of people over 60 years old expected to exceed 2 billion.^[7]^ As individuals age, their physical fitness and functional levels tend to decline, leading to the development of obesity, hypertension, cardiovascular disease, muscle atrophy, and osteoporosis. Among these conditions, dyslipidemia is recognized as a major risk factor for coronary heart disease, which can ultimately lead to death.^[8–14]^

Regular and adequate physical activity improves cellular activity, promotes the consumption of energy substances such as sugars and lipids, reduces risk factors for metabolic diseases, decreases the incidence of disease,^[15–17]^ inhibits a lesser decline in physical function, and reduces the risk of cardiovascular disease by positively influencing individuals with dyslipidemia.^[18]^

However, different forms of exercise have varying effects on the blood lipid levels in older adults. Several studies have shown that older adults who engage in aerobic exercise (AE) have a largely effective improvement in lipid levels compared to those who are physically frail,^[19,20]^ while resistance exercise (RE) and aerobic exercise are both effective in reducing blood lipid levels and ameliorating lipid profile in older adults.^[21]^ In addition, several studies have also shown that aerobic exercise combined with resistance exercise (AE+RE) and can decrease TC, TG, and LDL-C indices.^[22,23]^ Current evidence also suggests that high intensity interval training (HIIT) may offer a promising approach for improving the metabolic health of female populations, including those with type 2 diabetes, dyslipidemia, and dyslipidemia associated with hyperglycemia. HIIT has been shown to effectively reduce TC, TG, and LDL-C, while also increasing HDL-C in some female subjects.^[24]^

While exercise has many health benefits, it is unclear which types of exercise are most beneficial for ameliorating the lipid profile in the elderly population. Therefore, this study aims to compare the effects of different exercise modalities on lipid profiles in the elderly population through meta-analysis and provide a reference for selecting an appropriate exercise modality to maintain good lipid profile levels and reduce the risk of metabolism-related diseases in the elderly population.

## 2. Materials and methods

This study aimed to conduct a systematic review and meta-analysis to investigate the effects of various exercise modalities on lipid profiles among healthy elderly individuals. The study followed the PRISMA 2020 statement for conducting systematic reviews and utilized the PICOS framework for formulating the research question and study design.^[8,25]^

The PICOS framework included Population (healthy elderly subjects), Intervention (different exercise modalities), Control (healthy elderly subjects without training), Outcomes (TC, TG, LDL-C, and HDL-C), and Study design (published randomized controlled trials). The research question addressed in this study was “What are the effects of different forms of exercise on lipid profiles in the elderly population?.”^[26]^

### 2.1. Search strategy

This study was conducted in strict accordance with the Preferred Reporting Items for Systematic Reviews and Meta-Analyses guidelines.^[8,25]^ To locate relevant studies, we performed a comprehensive literature search using the following keywords: “elderly,” “exercise,” “total cholesterol,” “triglycerides,” “LDL cholesterol,” and “HDL cholesterol,” along with their respective synonyms, near-synonyms, and abbreviations. We searched multiple databases, including PubMed, EBSCO, Web of Science, China National Knowledge Infrastructure, and Wanfang database, and refined the search results for different types of exercise, such as “aerobic exercise (AE),” “resistance exercise (RE),” “aerobic combined resistance exercise (AE+RE),” and “high intensity interval training (HIIT).” We combined all search terms in various permutations to maximize the retrieval of relevant studies.

It’s important to note that we did not require all exercise categories to appear in conjunction with all the lipid profile markers, but rather considered studies that reported on any of the lipid profile markers of interest. To ensure the quality of the studies, we excluded gray literature studies during the search process and reviewed the reference lists of previously published relevant articles to identify additional studies.

Our search strategy employed a combination of subject terms and free words, covering studies from the creation of the database until December 2022. By tracking down relevant literature references, we aimed to identify all studies that met our inclusion criteria.

### 2.2. Selection and exclusion criteria of literature

*Inclusion criteria*: subjects: healthy subjects who were free of hypertension, diabetes, cardiovascular and other related diseases, with a mean age of 60 years or older, regardless of race and nationality; study type: published randomized controlled trials; intervention was exercise of any intensity, form and duration for at least 4 weeks; and outcome indicators included more than two of TC, TG, HDL-C, and LDL-C.

*Exclusion criteria*: adolescent literatures, reviews, conference papers, and dissertations with incomplete data ranges; individual case studies; the full text or the literature with unclear experimental data that could not be obtained; animal-based studies; and duplicate publications.

### 2.3. Studies selection and data extraction

The search results were merged and imported into Endnote X9 literature management software, where the abstract included the first author, year of publication, sample size, number of men and women, age, exercise training form, intervention modality (exercise pattern, period, time, frequency), and outcome evaluation indicators of the literature. The process of inclusion in this study was not restricted to any single language; rather, it involved a selection of languages deemed relevant to the investigation. Specifically, English, Chinese, and Korean were included in this study.

Duplicates were first removed, and then the titles and abstracts of the literature were initially screened according to the inclusion and exclusion criteria, respectively, to exclude those that did not meet the requirements, and inclusion for in the study was further determined after reading the full text. This process was carried out independently by 2 researchers (H.Y. and W.S.), and for literature that could not be identified for inclusion or exclusion, a negotiation between the 2 investigators was required to resolve the discrepancies, and a third researcher (H.Z.) was required to adjudicate if necessary.

Data extracted from the included literature include: first author and year of publication; sample size; age; intervention modality; intervention expectation; and outcome evaluation indicators. A detailed summary of the extracted date is provided in Table 1.

**Table 1:** General characteristics of selected research literature.

Authors nation	Year	Sample size (n)			Age (yr)	Duration of intervention (min)	Intervention	Frequency	Duration (wk)	Outcome
		Intervention group/BMI	Control group/BMI	Gender M:F						
Fox et al^[27]^ <America>	1996	16 30.6 ± 2.1	13 29.8 ± 1.5	(0):(29)	65.6 ± 3.3	60	AE+RE	5/W	24	①②③④
Cao et al^[28]^ <China>	2019	13 28.0 ± 2.9	15 26.4 ± 1.4	(0):(28)	64.2 ± 5.1	60	AE	3/W	12	①②③④
Valente et al^[8]^ <America>	2011	15	12	(11):(16)	66.6 ± 4.3	40	RE	3/W	10	①②③④
		25–39.9								
Park et al^[29]^ <South Korea>	2020	10 26.2 ± 0.5	10 26.0 ± 0.4	(20):(0)	68.8 ± 0.9	90–120	AE+RE	3/W	12	①②③④
Rezaeipour^[30]^ <Iran>	2021	28 35.4 ± 5.2	27 35.6 ± 5.4	(55):(0)	68.7 ± 3.2	60	HIIT	3/W	12	①③④
Shah et al^[31]^ <America>	2009	9	9	(5):(13)	≥65	90	AE+RE	3/W	24	①②③④
		≥30								
Tomeleri et al^[32]^ <Brazil>	2016	19 27.8 ± 4.5	19 27.1 ± 3.8	(0):(38)	≥60	45–50	RE	3/W	8	①②③④
Boardley et al^[33]^ <America>	2007	33 (AE) 29.8 ± 4.2 31 (RE) 31.1 ± 6.8 32 (AE+RE) 29.0 ± 5.3	35 29.4 ± 4.6	(36):(95)	74.6 ± 6	45–50	AE, RE, AE+RE	3/W	16	①②③④
Ha et al^[34]^ <South Korea>	2018	11 23.92 ± 1.82	9 29.11 ± 12.47	(0):(20)	70–80	50	AE	3/W	12	①②③④
Marinda et al^[35]^ <South Africa>	2013	25 28.32 ± 6.77	25 29.32 ± 5.44	(0):(50)	≥60	60	AE	3/W	8	①②
So et al^[36]^ <South Korea>	2013	18 25.9 ± 3.0	22 25.9 ± 2.8	(13):(27)	65–82	60	RE	3/W	12	①②③
Sunami et al^[37]^ <Japan>	1999	20 22.0 ± 2.3	20 22.7 ± 2.8	(20):(20)	60–77	60	AE	2–4/W	20	①②③④
Liu and Jinal^[38]^ <China>	2010	34	30	(0):(64)	65.7 ± 3.1	40–50	AE	6/W	24	①②③④
		Not described								
Wang et al^[39]^ <China>	2009	111	105	Not described	60–79	40–60	AE	3–4/W	48	①②③④
		Not described								
Zhang et al^[40]^ <China>	2016	20 24.05 ± 2.59	20 23.92 ± 2.57	(0):(40)	>60	60	AE	4/W	12	①②③④
Ballin et al^[7]^ <Sweden>	2019	36	36	Not de-scribed	70.7 ± 0.2	18–36	HIIT	3/W	10	①②③④
		29.2 ± 3.3								
Kim and Oh^[41]^ <South Korea>	2021	10	10	(0):(20)	>65	50	AE	3/W	12	①②③④
		Not described								
Kim and Cho^[42]^ <South Korea>	2013	10 (AE) 23.10 ± 1.07 10 (RE) 23.35 ± 3.22 10 (AE+RE) 24.07 ± 2.02	10 23.24 ± 2.97	(0):(40)	>65	70	AE, RE, AE+RE	3/W	12	①②③④
Sun^[43]^ <South Korea>	2017	12 24.28 ± 3.02	12 24.66 ± 2.56	(0):(24)	>80	60	AE	5/W	24	①②③④
Hun^[44]^ <South Korea>	2020	8 23.85 ± 2.95	8 24.70 ± 2.96	(16):(0)	66–77	60	AE	3/W	12	②③④

AE = aerobic exercise, AE+RE = aerobic+resistance exercise, HIIT = high-intensity interval training, M:F(male):(female), RE = resistance exercise, W = week(s), ① TC = total cholesterol, ② TG = triglyceride, ③ HDL-C = high-density lipoprotein cholesterol, ④ LDL-C = low-density lipoprotein cholesterol.

### 2.4. Quality assessment

The Cochrane Risk of Bias Tool version 2 was used to evaluate the risk of bias in the included studies. Cochrane Risk of Bias Tool version 2 assesses several components of study quality, including the randomization process, deviations from the intended intervention, missing outcome data, measurement of the outcome, and selection of the reported outcomes. This assessment was performed separately for each study in the analysis. According to the systematic evaluation manual, researchers classified articles as low risk, high risk, or some concerns. The assessment of risk of bias was completed independently by 2 reviewers (H.Y. and Y.S.), and conflicts were resolved by a third reviewer (C.L.). The present study utilized the Grading of Recommendations Assessment, Development, and Evaluation (GRADE) methodology to rigorously evaluate the quality of the evidence that was retrieved. This approach is widely recognized as a scientifically sound method for systematically appraising the quality of evidence in a given field. By using the GRADE methodology, the study was able to ensure that its conclusions were based on a thorough and objective evaluation of the available evidence.

### 2.5. Statistical analysis

Data were analyzed using RevMan5.4, with effect sizes expressed as weighted mean differences and 95% confidence intervals (CIs). Heterogeneity was analyzed using the consistency coefficients *I*^2^ and *P*. If *I*^2^ > 50% and *P* < .10 indicated heterogeneity between studies, the analysis was performed using a random-effects model; conversely, a fixed-effects model was used. Subgroup analyses were performed according to movement patterns. Sensitivity analyses were used to detect the effect of individual studies on the total effect. When statistical heterogeneity existed but there was no clinical heterogeneity between study groups, a random-effects model was used for analysis. If the heterogeneity was too pronounced to identify its source, only descriptive analyses were used. When the number of studies was more than 10, funnel plots were used to explore publication bias, and a significance level of α = 0.05 was chosen for the significance test of articles.

## 3. Results

### 3.1. General results of the selected research literature

**Figure 1.**
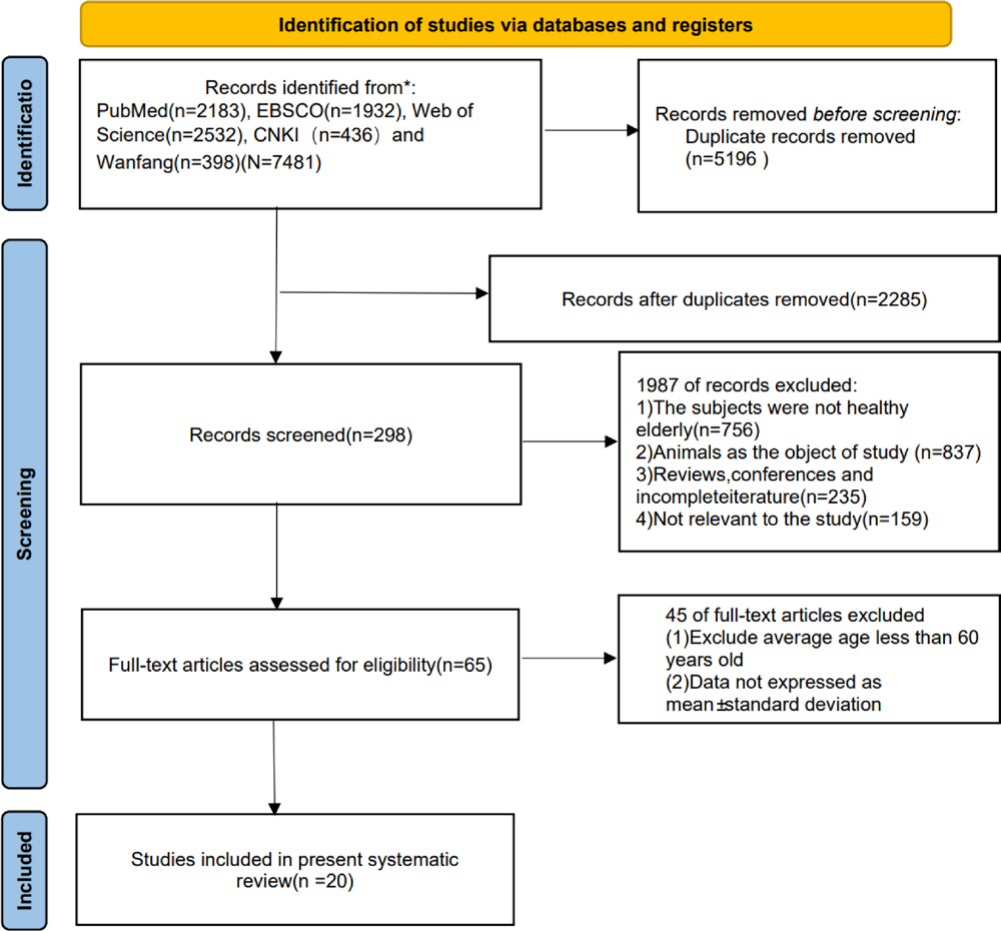
Flow chart of literature screening.

The study utilized a rigorous and systematic search strategy to identify relevant papers from the database, resulting in a total of 7481 papers. 5196 duplicate papers were removed using Endnote software, leaving 2285 papers for further screening. Subsequently, the titles and abstracts of the remaining papers were carefully assessed against predefined inclusion and exclusion criteria, leading to the exclusion of 1987 papers. Following this, a thorough evaluation of the remaining 298 papers was conducted, resulting in the selection of 20 studies for inclusion in the meta-analysis. A graphical representation of the selection process is provided in Figure 1. This stringent screening process employed in this study ensured that only high-quality studies meeting the established criteria were included in the meta-analysis.

### 3.2. General characteristics of the selected research literature

After conducting a thorough screening process, following pre-determined inclusion and exclusion criteria, 20 randomized controlled trials were selected for inclusion in this meta-analysis. These trials involved various forms of exercise interventions such as aerobic exercise, resistance exercise, aerobic combined with resistance exercise, and high-intensity intermittent training. The total number of subjects included in these trials was 988, with a mean age greater than 60 years. Further information regarding the selected trials can be found in Table 1.

### 3.3. Quality evaluation of the selected literature

**Figure 2.**
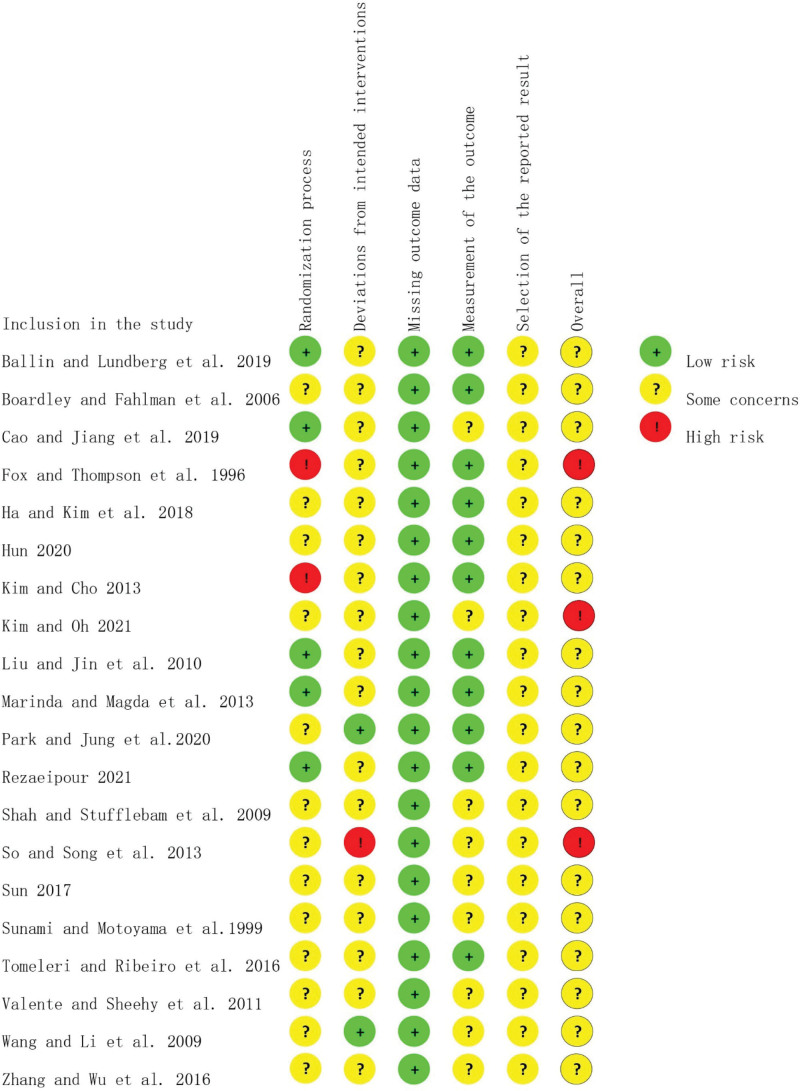
Risk of bias summary. + = low risk, ! = high risk, ? = some concerns.

An individualized risk of bias assessment was conducted for the included study literatures using the Cochrane Risk of Bias Tool version 2. The assessment resulted in 7 entries, which are illustrated in Figure 2. Since all interventions in this study were exercise-related, achieving complete double-blinding during the experiment was not feasible. Nevertheless, we ultimately included 20 papers in our analysis, with the remaining entries demonstrating low risk. It is noteworthy that only three out of the 20 papers were identified as having a high risk during the overall evaluation. Importantly, all of them were assessed as having a low risk in terms of missing outcome data.

### 3.4. Meta-analysis of the effect of different exercise modalities on the TC level in the elderly

The heterogeneity of the studies included in the analysis was relatively high, with an *I*^2^ value of 52% and a *P* value of .002. After combining the effect sizes using a random effects model, the standardized mean difference (SMD) was found to be −0.4, with a 95% CI of −0.59 to −0.21 (*P* < .01). These findings suggest that there were significant differences in TC levels among the different forms of exercise.

**Figure 3.**
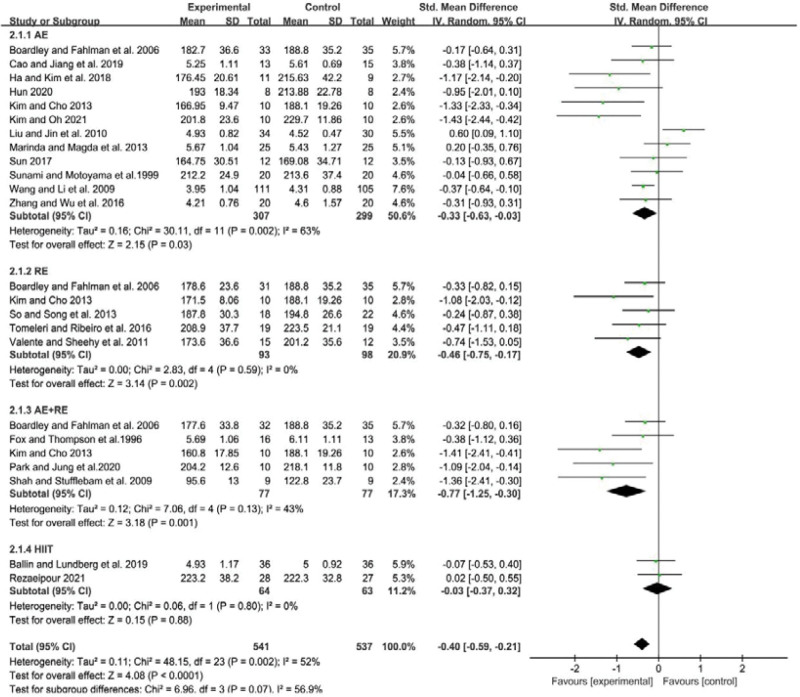
Meta-analysis of the effect of different exercise modalities on the TC level in the elderly. AE = aerobic exercise, AE+RE = aerobic+resistance exercise, HIIT = high-intensity interval training, RE = resistance exercise, TC = total cholesterol.

Further subgroup analysis showed that AE, RE and AE+RE all significantly reduced TC levels in older adults. Specifically, the SMD for AE was −0.33 (95% CI: −0.63 to −0.03, *P* < .05), for RE was −0.46 (95% CI: −0.75 to −0.17, *P* < .01), and for AE+RE was −0.77 (95% CI: −1.25 to −0.30, *P* < .01). However, there was no significant difference in the improvement of TC levels in older adults with HIIT (SMD = −0.03, 95% CI: −0.37 to 0.32, *P* = .88). The combined effect size was statistically significant, with AE+RE demonstrating a greater reduction in TC levels in older adults compared to the other 3 exercise modalities. These findings are illustrated in Figure 3.

In summary, these results suggest that AE, RE, and AE+RE are effective in reducing TC levels in older adults, while HIIT may not be as effective. These findings provide important insights for healthcare professionals when designing exercise interventions for older adults with high TC levels.

### 3.5. Meta-analysis of the effect of different exercise modalities on the TG level in the elderly

The level of heterogeneity among studies was assessed using the *I*^2^ statistic, which indicated a low degree of heterogeneity (*I*^2^ = 38%, *P* = .04). The combined effect sizes were calculated using a fixed effects model, which yielded a SMD of −0.28 with a 95% CI of −0.41 to −0.16 (*P* < .01). These findings suggest that there were significant differences in TG levels among the different forms of exercise.

**Figure 4.**
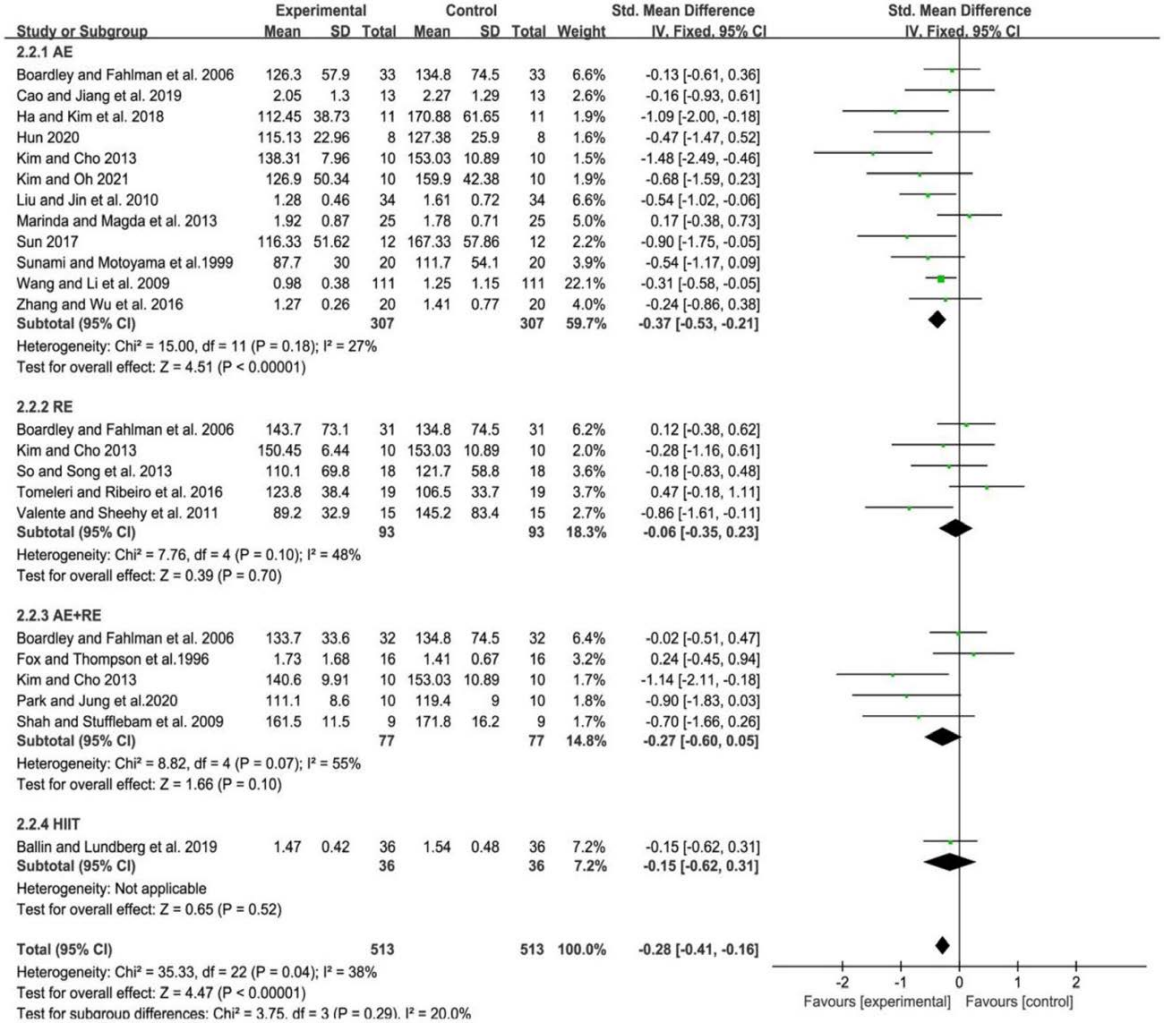
Meta-analysis of the effect of different exercise modalities on the TG level in the elderly. AE = aerobic exercise, AE+RE = aerobic+resistance exercise, HIIT = high-intensity interval training, RE = resistance exercise, TG = triglycerides.

Further subgroup analysis was conducted to investigate the effect of different types of exercise on TG levels in the elderly. The results indicated that AE was the only form of exercise that significantly reduced TG levels in older adults (SMD = −0.37, 95% CI = −0.53 to −0.21, *P* < .01). The other forms of exercise, namely RE, AE+RE, and HIIT, did not show a significant effect on TG levels in the elderly (*P* = .70, .10, .52, respectively). Specifically, RE had a non-significant effect on TG levels (SMD = −0.06, 95% CI = −0.35 to 0.23, *P* = .70), AE+RE had a non-significant effect on TG levels (SMD = −0.27, 95% CI = −0.60 to 0.05, *P* = .10), and HIIT had a non-significant effect on TG levels (SMD = −0.15, 95% CI = −0.62 to 0.31, *P* = .52). These findings are illustrated in Figure 4.

In summary, the results suggest that AE is an effective form of exercise for reducing TG levels in older adults. However, other forms of exercise did not show a significant effect on TG levels in this population. These findings have important implications for exercise prescription in older adults and may inform the development of targeted exercise interventions for the management of TG levels in this population.

### 3.6. Meta-analysis of the effect of different exercise modalities on the HDL-C level in the elderly

The analysis of the studies demonstrated a relatively substantial heterogeneity, as indicated by an *I*^2^ value of 79% and a *P* value of less than .01. After combining effect sizes using a random-effects model, we found that the difference in HDL-C levels between various forms of exercise was statistically significant, with a standardized mean difference of 0.55 (95% CI: 0.26–0.85; *P* < .01).

**Figure 5.**
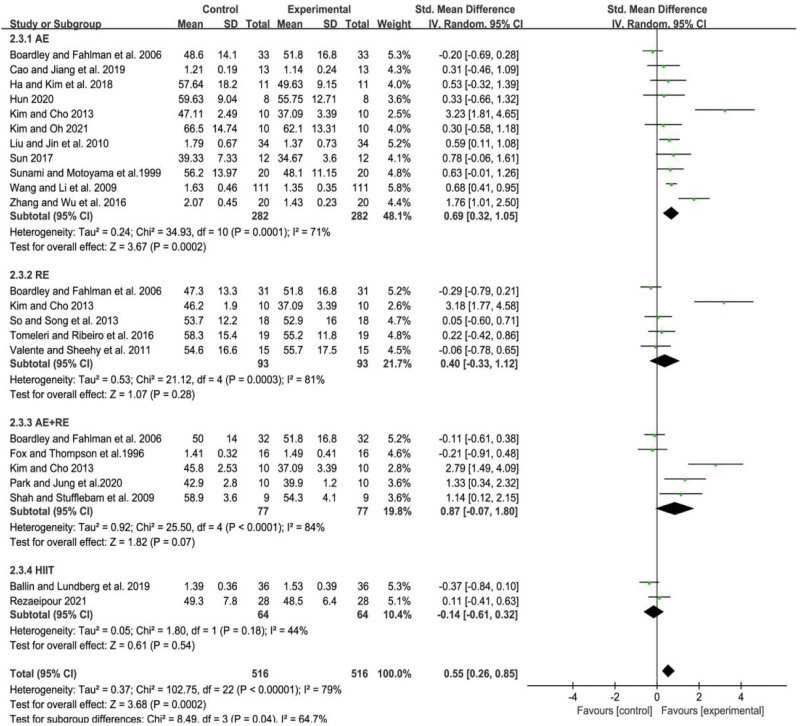
Meta-analysis of the effect of different exercise modalities on the HDL-C level in the elderly. AE = aerobic exercise, AE+RE = aerobic+resistance exercise, HDL-C = high-density lipoprotein cholesterol, HIIT = high-intensity interval training, RE = resistance exercise.

To investigate the effect of different types of exercise on HDL-C levels in older adults, a subgroup analysis was performed. The results revealed that only AE led to a significant improvement in HDL-C levels, with a standardized mean difference of 0.69 (95% CI: 0.32–0.05; *P* < .01). However, RE, AE+RE, and HIIT did not result in significant increases in HDL-C levels in older adults, with *P* values of .28, .07, and .54, respectively. These findings are presented in Figure 5, where RE had a non-significant effect on HDL-C levels (SMD = 0.40, 95% CI = −0.33 to 1.12, *P* = .28), AE+RE had a non-significant effect on HDL-C levels (SMD = 8.87, 95% CI = −0.07 to 1.80, *P* = .07), and HIIT had a non-significant effect on HDL-C levels (SMD = −0.14, 95% CI = −0.61 to 0.31, *P* = .54).

Therefore, the findings suggest that AE is an effective form of exercise for increasing HDL-C levels in older adults. However, other forms of exercise did not show a significant effect on HDL-C levels in this population. These results are important for exercise prescription in older adults and may inform the development of targeted exercise interventions for managing HDL-C levels in this population.

### 3.7. Meta-analysis of the effect of different exercise modalities on the LDL-C level in the elderly

The heterogeneity between studies was found to be relatively high (*I*^2^ = 78%, *P* < .01). A random effects model was used to combine the effect sizes, resulting in a statistically significant difference in the effects of different forms of exercise on LDL-C (SMD = −0.79, 95% CI = −1.10 to −0.49, *P* < .01).

Subgroup analysis was conducted to further investigate the effects of different exercise modalities on LDL-C. The results showed that AE had a significant LDL-C reduction effect (SMD = −0.49, 95% CI = −0.76 to −0.21, *P* < .01), as did RE (SMD = −1.75, 95% CI = −3.27 to −0.22, *P* < .05) and AE+RE (SMD = −1.17, 95% CI = −1.98 to −0.35, *P* < .01). However, HIIT did not have a statistically significant effect on LDL-C (SMD = −0.19, 95% CI = −0.90 to −0.52, *P* = .60).

**Figure 6.**
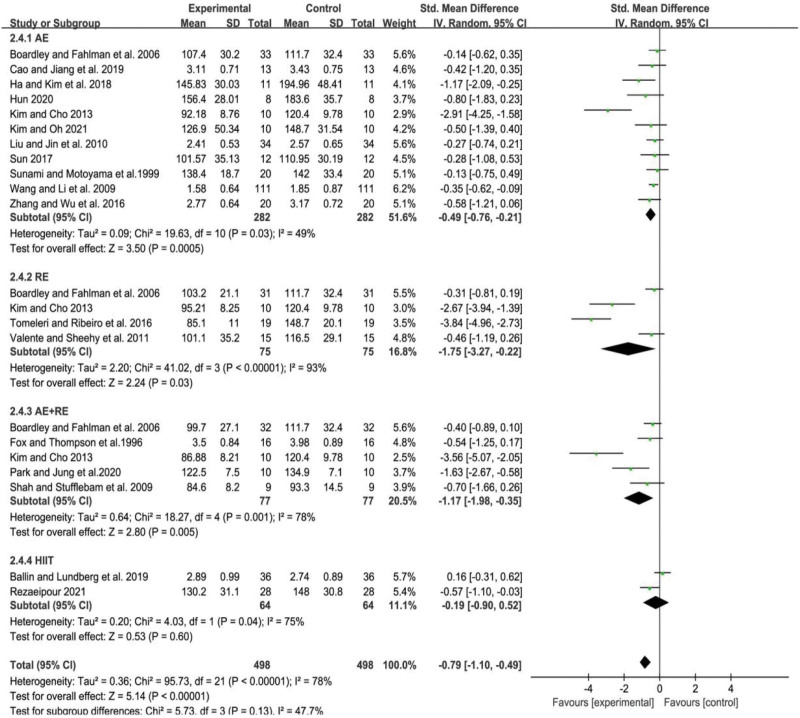
Meta-analysis of the effect of different exercise modalities on the LDL-C level in the elderly. AE = aerobic exercise, AE+RE = aerobic+resistance exercise, HIIT = high-intensity interval training, LDL-C = low-density lipoprotein cholesterol, RE = resistance exercise.

Notably, the results suggest that AE, RE, and AE+RE are effective in reducing LDL-C in older adults, while HIIT may not be as effective (Fig. 6). Therefore, it can be concluded that regular exercise, such as AE, RE, and AE+RE, may have significant beneficial effects on reducing LDL-C levels in older adults.

### 3.8. Publication bias and sensitivity analysis

**Figure 7.**
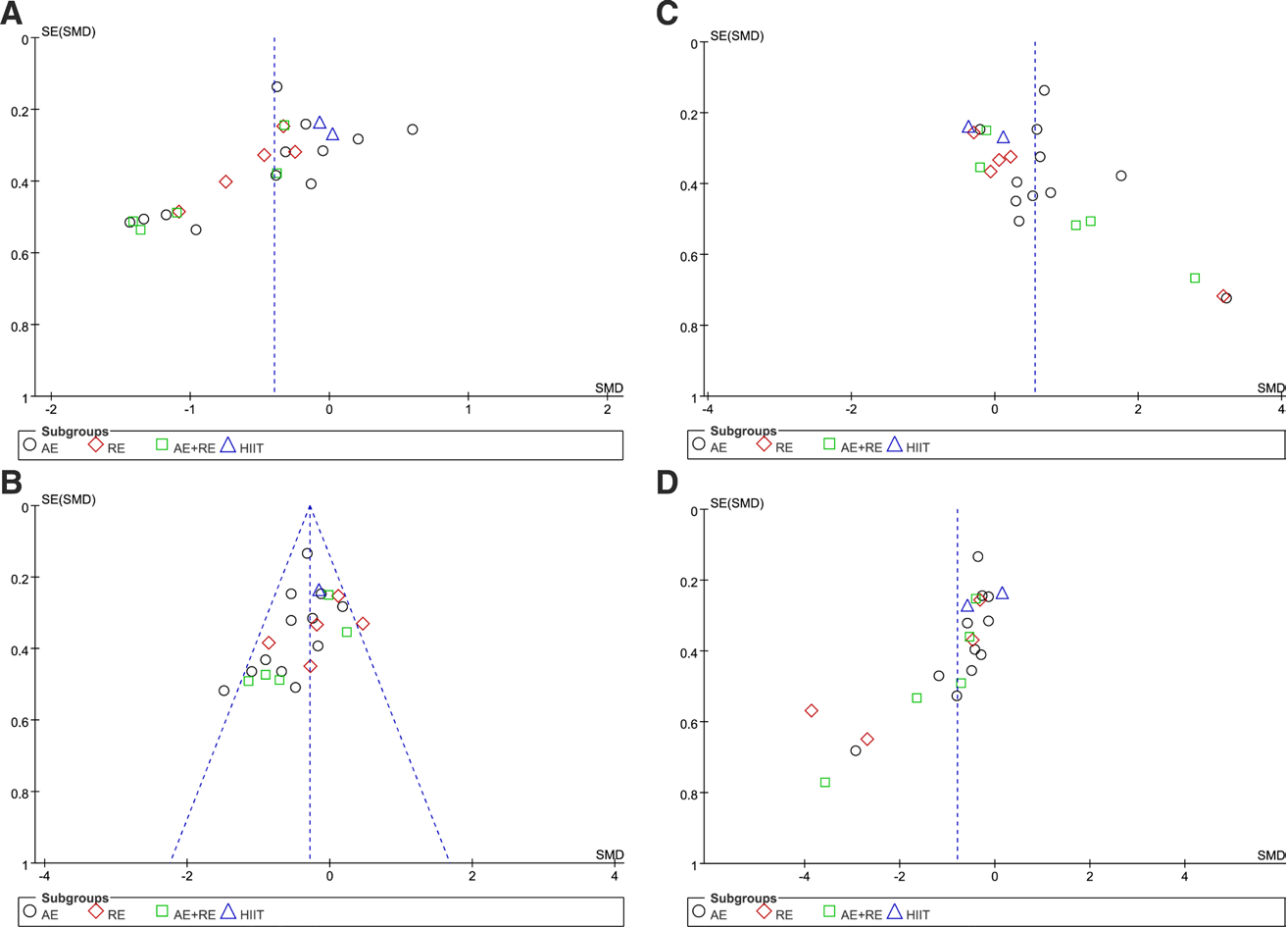
Publication bias of the influence of different exercise modalities on the TC (A), TG (B), HDL-C (C), and LDL-C (D) level in the elderly. HDL-C = high-density lipoprotein cholesterol, LDL-C = low-density lipoprotein cholesterol, TC = total cholesterol, TG = triglycerides.

Funnel plots were generated to assess the distribution of the indicators of TC, TG, HDL-C, and LDL-C. The findings revealed that the distribution of the funnel plot for TG was approximately symmetrical, while the funnel plots for TC, HDL-C, and LDL-C were predominantly asymmetrical (Fig. 7). To test the stability of the results, a sensitivity analysis was performed by altering the combined effect size and excluding individual studies before conducting the meta-analysis again. The outcomes demonstrated that the changes were not statistically significant compared to the previous results, signifying the stability of the meta-analysis findings and the reliability of the study’s conclusions.

### 3.9. Grading of Recommendations Assessment, Development, and Evaluation

**Figure 8.**
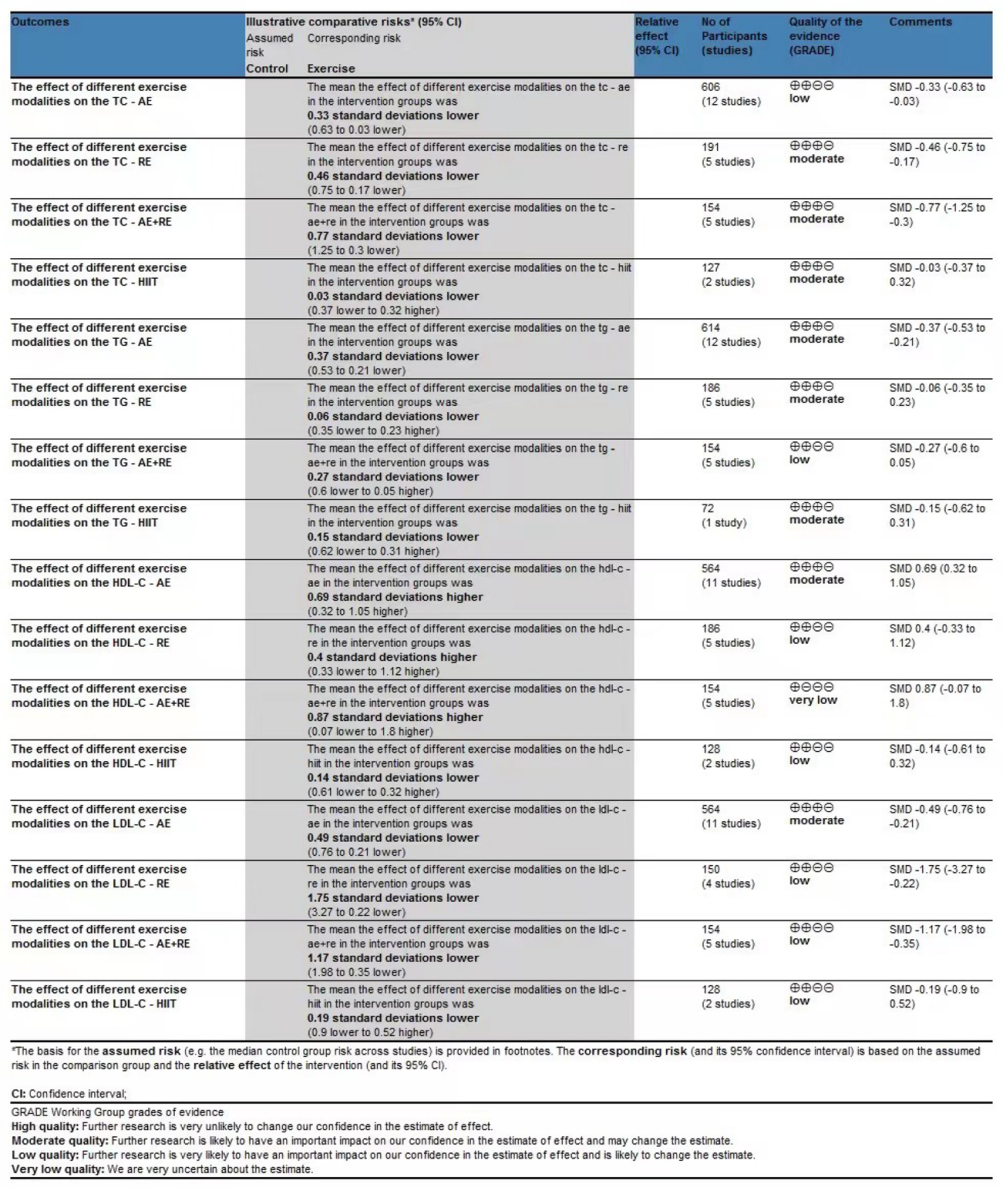
GRADE table for the influence of different exercise modalities on the TC, TG, HDL-C, and LDL-C level in the elderly. GRADE = Grading of Recommendations Assessment, Development, and Evaluation, HDL-C = high-density lipoprotein cholesterol, LDL-C = low-density lipoprotein cholesterol, TC = total cholesterol, TG = triglycerides.

The quality of evidence in the randomized controlled trial study was evaluated using the GRADE approach. The analysis of TC indicated moderate quality evidence for the RE, AE+RE, and HIIT groups. In the case of TG, the quality of evidence was moderate for the AE, RE, and HIIT groups. For HDL-C and LDL-C, the quality of evidence was moderate for the AE group. In addition, the quality of evidence for TG was moderate for the AE group (Fig. 8). The assessment of other outcomes in the study was downgraded to low due to various reasons, such as inadequate literature.

## 4. Discussion

The primary objective of this study was to investigate the impact of different exercise modalities on lipid profile in the elderly population. The results revealed that diverse exercise modalities could mitigate lipid profile abnormalities in the elderly population to a certain extent. Specifically, a 0.026 mmol/L increase in HDL-C was associated with a 2% reduction in the risk of coronary heart disease in men and at least 3% in women, while a 1% reduction in LDL-C led to a 2% to 3% decrease in the risk of coronary heart disease.^[45]^ Furthermore, TC is extremely important for cardiovascular health, as high blood TC levels are positively correlated with cardiovascular disease. Regular participation in exercise can lower blood TC levels, thus preventing cardiovascular-related diseases.^[14]^ Exercise training slows the decline of HDL-C while reducing dietary intake of saturated fat and cholesterol, and promoting the decline of LDL-C.^[45]^ A previous study^[46]^ reported that aerobic exercise improved plasma lipoprotein and lipid levels in older adults, that is, TC, TG, and LDL-C decreased significantly and HDL-C increased significantly, thereby reducing the risk of cardiovascular disease, which is consistent with the present study’s findings.^[46]^

Regular exercise has been shown to have a positive impact on lipid metabolism in skeletal muscle. One possible mechanism by which exercise improves lipid utilization is by increasing the activity of lecithin-cholesterol acyltransferase, an enzyme responsible for ester transfer from human plasma to HDL cholesterol.^[47]^ Additionally, exercise training has been found to enhance lipoprotein lipase activity, which plays a crucial role in lipid uptake by muscles.^[48]^

Research by Ferguson et al has indicated that a minimum energy expenditure of 1100 kcal during exercise is required to trigger a significant increase in HDL cholesterol levels, which is consistent with the observed increase in lipoprotein lipase activity.^[49]^ Furthermore, a study by Kraus et al investigated the effects of exercise volume and intensity on lipid levels and found that high exercise volume had a more significant positive impact on lipid profiles and the prevention of cardiovascular disease than low exercise volume.^[50]^ Specifically, the study found that training volume, rather than training intensity, was the key determinant of improvements in lipid levels.

Kraus et al conducted an 8-month aerobic exercise intervention with 111 randomized subjects divided into 3 intervention groups (walking 12 miles per week, jogging 12 miles per week, and jogging 20 miles per week) and a control group.^[50]^ The study found that higher levels of exercise had a much greater beneficial effect on lipid profiles than lower levels of exercise. Although research has shown that 10 weeks of HIIT as a single intervention can significantly reduce TC and LDL-C in centrally obese 70-year-old individuals,^[7]^ HIIT did not significantly ameliorate lipid profiles in older adults in our study. This may due to the limited literature on HIIT as more research is required to confirm these findings or due to insufficient energy expenditure, similar to the findings of Nybo et al.^[51]^

Under dietary education conditions, the RE intervention had a stronger tendency to lower TG and LDL-C than the no RE intervention, but no statistical difference was demonstrated.^[8]^ Resistance exercise and aerobic combined with resistance exercise modality showed significant changes only in TC and LDL-C, while aerobic exercise played a significant role in TC, TG, LDL-C, and HDL-C. Meanwhile, prolonged physical activity has been found to be closely associated with lipid profiles, and it requires sufficient training volume to induce changes in fat mass that favorably alter lipid profiles. Therefore, prolonged aerobic exercise may be more effective than the other 3 forms of exercise in improving blood lipids in the elderly.^[14]^

The research literature from China and Korea suggests that regular AE interventions can be effective in improving lipid profiles and overall cardiovascular health in older adults, particularly women over 60 years of age. Specifically, a 12-week, 4-day-a-week, 60-minute per day AE exercise intervention at 50% to 60% of maximum oxygen uptake can significantly reduce TC, TG, and LDL-C, while increasing HDL-C levels in older women.^[40]^

Additionally, other forms of exercise, such as ballet and elastic band training, have also been found to be effective in improving lipid profiles in older women. Specifically, 50 minutes of AE in the form of ballet 3 times a week for 12 weeks significantly helped older women lower TC, TG, and LDL-C and increase HDL-C.^[41]^ Four weeks of 70 minutes of combined aerobic exercise in elastic bands 3 times a week significantly helped older women lower TC, TG, and LDL-C, and increase HDL-C.^[42]^

Moreover, qigong, a traditional Chinese exercise that combines meditation, breathing, and movement, has also been found to have potential benefits for older women’s lipid profiles. Specifically, 24 weeks of qigong for 60 minutes 5 times a week significantly helped to lower TG in older women, but had no significant effect on changes in TC, LDL-C, and HDL-C.^[43]^ Meanwhile, dance sports exercise for 60 minutes, 3 times a week for 12 weeks, has also been found to be effective in helping older men lower TC, TG, and LDL-C.^[44]^ Overall, these findings suggest that regular exercise, particularly aerobic exercise, can be an effective intervention for improving lipid profiles and cardiovascular health in older adults.

Studies investigating the effects of different exercise modalities on blood glucose indicators in older adults have shown mixed results, with some studies reporting no significant improvements,^[31,36,37,52]^ while others have observed opposite results.^[29,32,36]^ Exercise is a cost-effective and practical form of health care that is superior to pharmacotherapy, but various factors such as exercise modality, intensity, duration, frequency of training, training environment, and temperature can all influence the outcomes of subjects’ blood indicators.^[46]^ Furthermore, the subjects’ status, such as obesity, hypertension, diabetes, and other comorbidities, can also impact the results of different studies.

The limited number of studies related to the effects of different exercise modalities on blood glucose indexes in the elderly population can lead to heterogeneity in the results, and future studies should focus on improving the quantity and quality of literature included. It is important to consider these factors when interpreting the results of studies investigating the effects of exercise on blood glucose indicators in older adults.

The present study utilized a rigorous meta-analysis approach, focusing exclusively on high-quality randomized controlled trials. To the best of our knowledge, this is the first meta-analysis to investigate the effects of exercise on glycolipid metabolism in the elderly population. The results indicate that exercise interventions of various types can improve lipid levels in older adults, with aerobic exercise being particularly effective. These findings have important implications for promoting exercise as a means of reducing glycolipid disorders in the elderly population. However, given the wide range of aerobic exercise modalities available, future research is needed to determine the optimal type and duration of exercise required to achieve the greatest benefits for older adults seeking to improve their lipid profiles.

## 5. Conclusions

According to scientific studies, various types of exercise can mitigate lipid profiles in elderly individuals. However, among the different forms of exercise, aerobic exercise has been found to yield better overall results. This conclusion is particularly true for older adults who have no prior experience with exercise training or are just starting to engage in physical activity. Therefore, it can be concluded that for this demographic, aerobic exercise is a more effective option for ameliorating lipid profiles.

## 6. Limitations of the study

Owing to the varied exercise intensities and patterns used in this study, we were unable to conduct a subgroup analysis to identify the most suitable exercise intensity and pattern for aerobic exercise. The current literature on HIIT as an exercise regimen for older adults is limited, and there may result in greater variability in the study outcomes. Additionally, the subjects selected for this study were healthy elderly individuals without any underlying health conditions, and we did not differentiate them based on gender or race. Moreover, significant heterogeneity may have affected our study due to variations in regimens, doses, durations, center settings, and enrollment populations.

## Acknowledgments

The first author (H.Y.) would like to express sincere gratitude to all the participants in this study. Additionally, the first author wishes to express his utmost appreciation to his family for their unwavering support, affection, and encouragement that have enabled him to successfully complete this research endeavor. Furthermore, the author would like to extend his acknowledgements to Dr Chang Liu from Beijing Sports University for providing invaluable feedbacks and meticulously reviewing the manuscript.

## Author contributions

**Conceptualization:** Wenbo Su, Haotian Zhao, Yaowei Sun, Chang Liu.

**Data curation:** Zhongjie Wang, Xianyou Cui, Changjin Xi.

**Software:** Wenbo Su, Huixin Li.

**Visualization:** Ruirui Gao.

**Writing – original draft:** Hezhang Yun.

**Writing – review & editing:** Hezhang Yun, Wenbo Su, Haotian Zhao, Yaowei Sun, Chang Liu.
